# Effects of bone marrow mesenchymal stem cell transplantation on tumor necrosis factor-alpha receptor 1 expression, granulosa cell apoptosis, and folliculogenesis repair in endometriosis mouse models

**DOI:** 10.14202/vetworld.2021.1788-1796

**Published:** 2021-07-10

**Authors:** Sri Ratna Dwiningsih, Soehartono Darmosoekarto, Hendy Hendarto, Erry Gumilar Dachlan, Fedik Abdul Rantam, Sunarjo Sunarjo, I. W. Arsana Wiyasa, Widjiati Widjiati

**Affiliations:** 1 Department of Obstetrics and Gynecology, Faculty of Medicine, Universitas Airlangga, Surabaya, Indonesia; 2 Department of Veterinary Science, Faculty of Veterinary Medicine, Universitas Airlangga, Surabaya, Indonesia; 3 Department of Obstetrics and Gynecology, Faculty of Medicine, Universitas Brawijaya Malang, Jl. Mayjen Prof. Dr. Moestopo No. 47, Surabaya 60132, Indonesia

**Keywords:** apoptosis, bone marrow mesenchymal stem cells, endometriosis, folliculogenesis, granulosa cells, tumor necrosis factor-alpha receptor 1

## Abstract

**Background and Aim:**

Endometriosis affects the ovaries and causes a decrease in the oocyte quality during endometrial receptivity. During the development of ovarian follicles, paracrine communication occurs between granulosa cells and oocytes. This study was conducted to determine the effects of bone marrow mesenchymal stem cell transplantation on tumor necrosis factor-alpha (TNF-α) receptor 1 (TNFR1) expression, granulosa cell apoptosis, and folliculogenesis in endometriosis mouse models.

**Materials and Methods:**

This study involved 42 female mice, which were divided into three groups: Healthy mice (T0), endometriosis mice without transplantation (T1), and endometriosis mice with bone marrow mesenchymal stem cell transplantation (T2). The mice were injected intraperitoneally with endometrial fragments (200 μL) to become endometriosis models. On day 15, the endometriosis models received mesenchymal stem cells. Sample collection was performed on day 29. Granulosa cell apoptosis and TNFR1 expression were examined using immunohistochemical staining, and folliculogenesis was assessed using hematoxylin and eosin staining of ovary samples. The data obtained from both examinations were statistically analyzed using Statistical Package for the Social Sciences.

**Results:**

The results showed that TNFR1 expression is significantly decreased in T2 (p<0.004). The apoptosis of granulosa cells was lower in T2 (p<0.000). The primary, secondary, and graafian follicle counts in T2 were significantly increased.

**Conclusion:**

Bone marrow mesenchymal stem cell transplantation in endometriosis mouse models can reduce TNFR1 expression and granulosa cell apoptosis and improve folliculogenesis.

## Introduction

Endometriosis is a gynecological disease that is commonly found in women of the reproductive age. The success rate of pregnancy in women with endometriosis who underwent *in vitro* fertilization (IVF) was 54% compared with infertile women due to tubal abnormalities [1]. Compared with other causes of infertility (e.g. tubal abnormalities and unexplained infertility), moderate endometriosis results in low pregnancy success and a high incidence of abortion [2]. Endometriosis has a negative impact on all markers of the infertility process in women who are participating in IVF programs [1], so there is a suspicion that endometriosis has a negative impact on the functions of the ovaries and Fallopian tubes and the ability of the uterus to accept conception [3]. Observations on IVF results using oocyte donors have proven the suspicion that endometriosis affects the ovaries and decreases the quality of oocytes during endometrial receptivity [4]. However, until now, the exact mechanism of folliculogenesis disturbance, which decreases oocyte quality in infertile patients with endometriosis, remains unclear.

The latest consensus on the pathogenesis of endometriosis has stated that endometriosis is a chronic inflammatory process in the pelvis that increases the function of immunological cells in the peritoneal fluid, which is unusual and is closely related to the growth and development of endometriosis, and affects the quality of the oocytes produced [5-9]. Endometriosis could also increase the apoptosis of granulosa cells; thus, it can affect the process of folliculogenesis and steroidogenesis [10]. The concentrations of pro-inflammatory interleukins (ILs) (i.e. IL-6, IL-1β, and IL-10) and tumor necrosis factor-alpha (TNF-α) were increased in patients with endometriosis. TNF-α concentration in the follicular flow of patients with endometriosis who have poor oocyte quality [3] is thought to trigger the secretion of other pro-inflammatory cytokines, which ultimately interfere with the process of oocyte fertilization. TNF-α plays a biological role as a pro-inflammatory, anti-tumor, and apoptotic agent through activated caspase-8, which activates caspase-3, and the apoptotic pathway begins [11].

Some previous studies [12,13] using stem cells to determine a decrease in apoptosis of granulosa cells in the ovaries of experimental animals given cytostatic have been conducted. These studies have shown the success of stem cell therapy for improving ovarian function. In this study, bone marrow mesenchymal stem cells were used because they have been widely used for therapy. Besides, bone marrow mesenchymal stem cells are a type of adult stem cell; thus, they have a small chance of becoming tumors compared with embryonic stem cells.

This study aimed to determine the effects of bone marrow mesenchymal stem cell transplantation on tumor necrosis factor-alpha (TNF-α) receptor 1 (TNFR1) expression, granulosa cell apoptosis, and folliculogenesis in endometriosis mouse models. , We used the severe combined immunodeficiency (SCID) endometriosis model (which has reduced immune function) as the study sample due to research ethics constraints.

## Materials and Methods

### Ethical approval

This type of study is explanatory research with pure experiments (true experiments) carried out in the experimental animal laboratory. This research has obtained ethical eligibility with ethics certificate number: 685-KE-2018.

### Study period and location

The study was carried out from May to July 2017 at the Stem Cell Laboratory of the Institute of Tropical Disease, Universitas Airlangga, Faculty of Veterinary Medicine, Universitas Airlangga and Cellular Biology Laboratory, Faculty of Mathematics and Natural Sciences, Universitas Brawijaya.

### The production of bone marrow mesenchymal stem cells

Bone marrow stem cells were obtained from female mice aged 3 months with a weight of 20–40 g. Under local anesthesia, the bone marrow aspirate was collected from the tibia using 5–10 mouse donors to reach 3 mL bone marrow and then placed into a 15 mL heparin tube containing 3 mL minimum essential medium-alpha (ratio 1:1) (Invitrogen, USA). The tubes were stored in the refrigerator before service. Each aspirate was transferred into a 15 mL blue cap tube and diluted with phosphate-buffered saline (PBS) (Sigma, USA). Each tube was processed with 5 mL PBS twice. Then, each aspirate was mixed and coated with Ficoll (G.E Healthcare, UK). The tubes were centrifuged at 1600 rpm for 15 min; then, the buffy coat located on Ficoll PBS was collected using a Pasteur pipette, and the cells were placed in a 15 mL tube. After that, each sample was diluted with PBS, mixed 3-5 times, and centrifuged again at 1600 rpm for 10 min. The supernatant was aspirated, and the cells were resuspended with 6 mL completed culture medium (CCM) (Invitrogen, USA). The cells were placed on a 5 cm^2^ plate and incubated with 5% CO_2_ humidity for 24 h. After 24 h, 2 mL PBS was added to the culture, which was washed twice. Then, 10 mL CCM was added, and the culture was incubated at 37°C humidity 5% CO_2_ for 5-10 days. Daily examinations were performed using an electron microscope. Every 3 days, the cells were washed with PBS 5% or 10%, and 10 mL CCM was added. The process was continued until the cells were between 60% and 80% confluent. Then, the passage is performed every 5 days until it reached passage four. After the stem cells from the bone marrow have been isolated, the next step is characterization to identify these stem cells. Stem cell identification through the genotypic approach using specific primers is known as the polymerase chain reaction approach. In preparing the stem stage, the expression of negative CD45 and positive CD73, CD90, and CD105 was characterized to ensure that the stem cells inserted were mesenchymal [14-18].

### Experimental animal preparation

Female mice aged 3 months with a weight of 20-40 g were adapted for 1 week in a clean cage with enough air, enough light, and adequate and homogeneous eating and drinking. The mice were randomly divided into three groups, each of which consisted of 14 mice: T0 group: Negative control group; T1 group: The positive control group (the group of endometriosis mouse models that received placebo); and T2 group: The treatment group (endometriosis mouse models that received bone marrow mesenchymal stem cells) [19].

### The production of endometriosis mouse models

The endometriosis model group of mice was intramuscularly injected with cyclosporine A (Sandimmune; Novartis, Basel, Switzerland) at a dose of 10 mg/body weight and estrogen. Endometriosis modeling using female mice aged approximately 12 weeks, weighing 20-40 g with 1 week of adaptation was performed by intramuscularly injecting 0.2 mL/mice cyclosporin A (Sandimmune; North Ryde, Australia), intraperitoneally injecting endometrial isolate (0.1 mL of human endometrial tissue has been transferred/injected to SCID mice), and intramuscularly injecting estrogen (a conversion dose of 5.4 mgr for each mouse [1 mgr equivalent to 10 IU]) on day 1. Estrogen injection was continued from day 2 until day 5. On day 14, the endometriosis model was complete, which was characterized by the growth of endometriosis tissue in the peritoneum. A single dose of bone marrow mesenchymal stem cells was administered on day 15 with a dosage of 10^6^/mice. On day 29, sample collection was performed [19,20].

### The examination of TNF-α receptor 1 (TNFR1) expression using immunohistochemical techniques

The sliced ovarian tissue was deparaffinated (paraffin block) with xylene thrice for 3 min each. Rehydration preparations were performed using 100% ethanol, 95% ethanol, and 70% ethanol for 2 min, 2 min, and 1 min, respectively, and finally using water for 1 min and then soaked in a peroxidase blocking solution at 27ºC for 10 min. The preparations were incubated in a pre-dilution inhibitor serum at 25°C for 10 min, soaked in 25°C monoclonal anti-TNFR1 antibodies (ab58436) for 10 min, and washed with PBS for 5 min. The preparations were incubated with secondary antibodies (conjugated with horseradish peroxidase) at 25°C for 10 min and then washed with PBS for 5 min. After that, they were incubated with 25°C peroxidase for 10 min and washed with PBS for 5 min. The preparations were incubated with diaminobenzidine at 25°C for 10 min, incubated with hematoxylin and eosin (H and E) for 3 min, and washed with running water. Then, they were cleaned, dropped with mounting media, and then covered with a coverslip. The expression of TNFR1 (brown in color) observed on the cells using a light microscope with 400×, which was previously confirmed at 1000×. All these examinations used a Nikon H600L light microscope (Tokyo, Japan) equipped with a 300 megapixel DS Fi2 digital camera and the Nikon Image System image processing software (Tokyo Japan). The semi-qualitative assessment of TNFR1 expression is based on Table-1 [21,22].

**Table-1:** The IRS semi-quantitative scale is the result of multiplying the positive cell percentage score (A) and the color reaction intensity score (B), IRS = (A × B).

A	B
Score 0: No positive cells	Score 0: No color reaction
Score 1: Positive cells <10%	Score 1: Low color intensity
Score 2: Positive cells between from 11%to 50%	Score 2: Medium color intensity
Score 3: Positive cells between from 51%to 80%	Score 3: High color intensity
Score 4: Positive cells over than 80%	

IRS=Immunoreactive score

### The examination of apoptosis cell expression using terminal deoxynucleotidyl transferase dUTP nick end labeling (TUNEL) techniques

Paraffin block sections were dewaxed; then rehydrated with 100% ethanol, 95% ethanol, and 70% ethanol and finally with water for 1 min; and then soaked in a 20 g/mL proteinase K (Sigma, St. Louis, MO) at 27ºC for 15 min. The tissues were incubated with a solution containing 2% H_2_O_2_ in PBS to inhibit endogenous peroxidase activity and then washed with PBS. The preparations were incubated in a terminal deoxynucleotidyl transferase (TdT) buffer solution containing 0.3 U/l TdT (Oncor, Gaithersburg, MD, USA), and 0.04 nmoL/L digoxigenin-dUTP (Oncor, Gaithersburg, MD, USA) was added to cover the tissues at 37°C for 60 min. Then, the tissues were washed with 300 mm sodium chloride and 30 mm sodium citrate for 30 min and then washed with PBS for 5 min. The preparations were incubated with anti-digoxigenin-peroxidase complex for 30 min at 27^o^C and stained with a solution of diaminobenzidine at 27°C for 10 min. The sections were counterstained with hematoxylin. Negative controls were obtained by omitting TdT from the buffer solution. The preparations were cleaned and dropped with mounting media and then covered with a coverslip. The expression of apoptosis cells (dark brown color) was observed using a light microscope with 400×, which was previously confirmed at 1000×. The semi-qualitative assessment of apoptosis cell expression is based on Table-1 [23,24].

### The examination of follicular development using H and E staining

The sliced ovarian tissue was deparaffinated with xylene thrice for 3 min each. The preparations were rehydrated using 100% ethanol, 95% ethanol, and 70% ethanol and finally using water for 1 min and then soaked in a peroxidase blocking solution at 27ºC for 10 min. The preparations were incubated with H and E for 3 min and washed with running water. Then, the preparations were cleaned and dropped with mounting media and covered with a coverslip. Follicular development was observed on the cells using a light microscope with 400×, which was previously confirmed at 1000× [25,26].

### Statistical analysis

For the difference analysis between the negative control, positive control, and treatment groups, one-way analysis of variance (ANOVA) was used in normally distributed data, and the Kruskal–Wallis test was used if the distribution was not normal. The expression of TNFR1 and apoptosis of granulosa cells were analyzed using ANOVA and *post hoc* test because the distribution was normal. The number of primary, secondary, tertiary, and graafian follicles was determined and examined using the Kruskal–Wallis test because the distribution was not normal.

## Results

This study consisted of three stages: Stem cell preparation, endometriosis model production, and proving the effects of mesenchymal stem cell transplantation on TNFR1 expression, granulosa cell apoptosis, and folliculogenesis repair. In the stem cell preparation stage, the expression of negative CD45 and positive CD73, CD90, and CD105 was characterized to ensure that the stem cells transplanted were mesenchymal. Then, to prove that the stem cells that we transplanted were homing into the ovary, we looked at PKH26 luminescence. In the second stage, to prove the evidence of the endometriosis model, we euthanized the seven mice in the T1, T2, and T0 groups on day 14.

### Isolation and culture of bone marrow mesenchymal stem cells of mice

**Figure-1:**
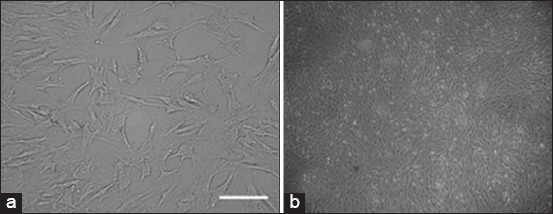
*Rattus norvegicus* bone marrow stem cell culture. (a) Mesenchymal stem cell morphology. Cells appear to be small cell bodies (fibroblast shaped), which are long and flattened with a large nucleus; (b) swirling pattern in passage four (inverted microscope, 40×).

Stem cells were collected from bone marrow aspirates from the tibia bone of the mice, and then, the stem cells were isolated according to the procedure in the stem cell laboratory of the Institute of Tropical Disease, Universitas Airlangga. When the cells were 80% confluent, half of the cells were planted back into a new Petri dish with the same medium for expansion. The passage is performed every 5 days until it reaches passage four. An overview of bone marrow stem cell culture is shown in Figure-1.

### Characterization of bone marrow mesenchymal stem cells

**Figure-2:**
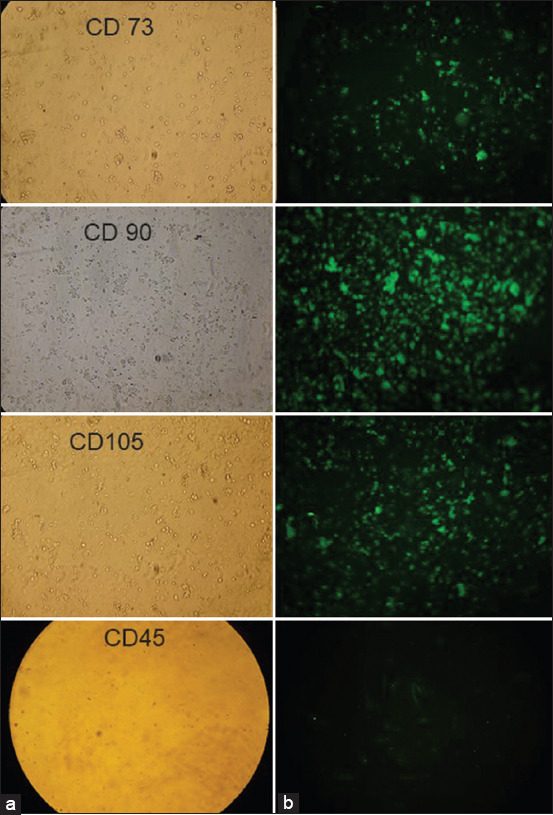
Immunocytochemical examination of CD 73, CD90, CD105, and CD 45. (a) Observation of bone marrow mesenchymal stem cells without fluorescence; (b) fluorescent observation of bone marrow mesenchymal stem cells (fluorescent microscope, 100×).

Stem cells developed *in vitro* can be identified using genotype (nucleotide) and phenotypic approaches. *In vitro* stem cells were characterized based on phenotypes by the immunocytochemical approach, using monoclonal or polyclonal antibodies that are labeled with fluorescence isothiocyanate (FITC) (F3651; Sigma, St. Louis, MO). In this study, mesenchymal stem cells were phenotypically identified by the immunocytochemical approach using monoclonal antibodies labeled with FITC. The cell markers CD73, CD90, and CD105 were checked to confirm mesenchymal stem cells and CD45 to remove bone marrow mesenchymal stem cells (Figure-2).

**Figure-3:**
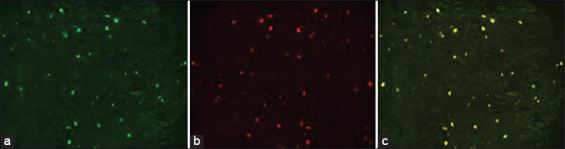
PKH26 luminescence in ovarian preparations of mice with endometriosis labeled PH26. (a) Green filter, (b) red filter, (c) red-green filter (fluorescent microscope, 4.2×).

Using a fluorescence microscope, immunofluorescence examination results showed a positive expression of CD73 (Figure-2). CD73 is a marker of mesenchymal stem cells. CD90 is also a marker of mesenchymal stem cells, and in this study, the cultured stem cells showed positive CD90 expression. No green luminescence was observed because the cultured stem cells did not express CD45 (Figure-2). PKH26 luminescence was observed in the bone marrow mesenchymal stem cell membrane (Figure-3), so the cells emitted red when observed under a fluorescence microscope. This is solid evidence that bone marrow mesenchymal stem cells can be homing in the ovarian tissue.

### Immunohistochemical results of TNFR1

**Figure-4:**
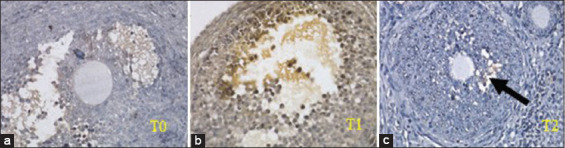
Comparison of R1 tumor necrosis factor-alpha expression in ovarian granulosa cells (arrows) among groups T0, T1, and T2 (immunohistochemistry stain, 1000×; Nikon H600L microscope; 300 megapixel DS Fi2 camera).

Immunohistochemical examination was intended to determine the expression of TNFR1 in the ovaries. The data resulting from this examination were semi-quantitative, assessed according to the modified Remmele method [27]. The Remmele scale index (immunoreactive score [IRS]) is the result of multiplying the percentage score of immunoreactive cells with the color intensity score on immunoreactive cells (Figure-4). The data for each sample are the average of the observed IRS value at five fields of view at 400×, which has been previously confirmed at 1000×.

After obtaining the IRS means, the data normality test was then conducted. Because the data were normally distributed, to determine the difference in the TNFR1 variable between groups, one-way ANOVA was used.

The expression of TNFR1 was significantly different among the three groups (p<0.05), so a further statistical examination was conducted using ANOVA. The *post hoc* test was used in this study because the variants of the three groups were not homogeneous. The *post ho*c test showed that the T1 group was significantly different from the T0 group (p<0.038) and the T2 group (p<0.017), but no significant difference was observed between the T0 and T2 groups (p>0.626) (Table-2). This study showed that endometriosis could increase TNF- R1 expression (T1) and administration of stem cells (T2) could reduce TNF- R1 expression similar to the control group (T0).

**Table-2:** *Post hoc* analysis of TNF-α R1 expression between groups.

Group	p-value
T0 group versus T1 group	0.038
T0 group versus T2 group	0.626
T1 group versus T2 group	0.017

T0 group: Negative control group; T1 group: The positive control group; and T2 group: The treatment group. TNF-α=Tumor necrosis factor-alpha

### Granulosa cell apoptosis

**Figure-5:**
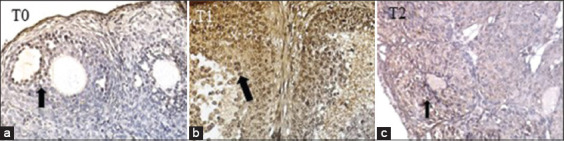
The difference in the number of ovarian apoptotic granulosa cells (arrows) between treatment groups (Tunel assay, 400×; Nikon H600L microscope; 300 megapixel DS Fi2 camera).

To determine the expression of apoptosis in granulosa cells of the ovarian follicles of mice, a histopathological examination was performed. The data for each sample were assessed semi-quantitatively according to the modified Remmele method (Figure-5). All these examinations used a Nikon H600L light microscope equipped with a 300 megapixel DS Fi2 digital camera and the Nikon Image System image processing software.

The IRS apoptosis data after the normality test showed that the data are normally distributed, so the data were analyzed using ANOVA. The apoptotic values of the three groups were significantly different (p<0.05), so a further statistical analysis using ANOVA was necessary. The *post hoc* test showed that the T0 group was significantly different from the T1 group (p<0.00), but no significant difference was observed between the T0 and T2 groups (p>0.19). Meanwhile, a significant difference was observed between the T1 and T2 groups (p<0.00) (Table-3).

**Table-3:** Apoptosis between the control group, the endometriosis model group, and the endometriosis model group of mice that were given with mesenchymal stem cells.

Group	n	Mean±Standard deviation	p-value
T0 group	14	2.54±1.462^a^	<0.0001
T1 group	14	6.39±2.317^b^	
T2 group	14	3.41±1.206^a^	

*Different superscripts show significant differences. T0 group: Negative control group; T1 group: The positive control group; and T2 group: The treatment group

### Follicle count result

To determine the number of primary, secondary, tertiary, and graafian follicles in the ovaries of mice, H & E staining was performed. The data on each sample were quantitative, where the number of follicles was counted in 400×. All these examinations used a Nikon H600L light microscope equipped with a calibrated micrometer, Nikon Image System image processing software, and a 300 megapixel DS Fi2 digital camera.

The statistical test results on the number of primary and secondary follicles between the three groups were not significantly different (p>0.05). The grade of folliculogenesis (tertiary follicles) among the three groups was not significantly different (p>0.05), whereas the graafian follicles showed significant differences among the three groups (p<0.05). The results of *post hoc* test on the graafian follicles showed that the T0 group was not significantly different from the T2 group (p>0.146), but a significant difference was observed between the T0 and T1 groups (p<0.005). Meanwhile, the number of graafian follicles between the T1 and T2 groups was significantly different (p<0.009) (Tables-4 and 5).

**Table-4:** The comparison of the number of primary and secondary follicles between groups.

Folliculogenesis	Group	n	Mean±Standard deviation	p-value
Primary follicle	T0 group	14	10.57±4.502^a^	0.222
	T1 group	14	7.71±3.024 ^a^	
	T2 group	14	10.00±5.657 ^a^	
Secondary	T0 group	14	4.93±3.772 ^a^	0.639
	T1 group	14	4.21±1.929 ^a^	
	T2 group	14	5.43±4.071^a^	

T0 group: Negative control group; T1 group: The positive control group; and T2 group: The treatment group

**Table-5:** The results of the statistical test on folliculogenesis (tertiary follicles and graafian follicle) between groups using the Kruskal–Wallis test.

Folliculogenesis	Group	n	Median (min-max)	p-value
Tertiary follicle	T0 group	14	2.5 (0-6)^a^	0.217
	T1 group	14	2.0 (1-2)^a^	
	T2 group	14	2.0 (0-3)^a^	
Graafian follicle	T0 group	14	4.0 (0-9)^a^	0.005
	T1 group	14	1.0 (1-3)^b^	
	T2 group	14	3.5 (0-5)^a^	

*Different superscripts show significant differences. T0 group: Negative control group; T1 group: The positive control group; and T2 group: The treatment group

## Discussion

The results of characterization in this study showed that the expression of CD73, CD90, and CD105 was positive, proving that stem cells originating from the bone marrow after culture grew into mesenchymal stem cells. The characterization test was performed to prove that the cultured stem cells are true mesenchymal stem cells using at least three antibodies (i.e. CD73, CD90, and CD105); this is to ensure that what grows is mesenchymal stem cells. The results of the study showed that some mesenchymal stem cells grow (Figures-2-4). Meanwhile, CD45, which is an antibody that can identify hematopoietic stem cells, was used as a positive control. In this study, the cultured stem cells did not express CD45 (Figure-2) [28-30].

Mesenchymal stem cells are multipotent cells whose capacity is functionally determined based on their ability to renew themselves and differentiate into several cell types. Mesenchymal stem cells can be isolated from various tissues, such as the bone marrow and skin. Markers on the surface of mesenchymal cells isolated from the skin, such as CD105, CD90, CD73, CD44, and CD200, can be found using immune fluorescence methods. Meanwhile, the presence of CD105, CD90, and CD73 can also be confirmed using flow cytometry [28-30].

The apoptosis of granulosa cells increases with the severity of the endometriosis. Granulosa cell apoptosis can also affect oocyte quality, which, in turn, will reduce the fertilization rate and reduce the pregnancy success rate [3]. The effects of endometriosis on granulosa cells have been described as change in steroidogenesis and cell cycle and increase in apoptosis. Endometriosis caused the dysregulation of molecular pathways in the development and growth of granulosa cells [31]. Endometriosis may alter various factors presented in follicular fluid by modifying the follicular oxidative stress status [32,33]. An increase in oxidative stress has been recently confirmed as the culprit of spindle disruption. Spindle disruption was an indicator of oxidative DNA damage, which leads to granulosa cell apoptosis [34]. Endometriosis also altered intrafollicular levels of pro-inflammatory cytokines. Follicular fluids from follicles aspirated from patients with endometriosis showed significantly higher concentrations of IL-8 and IL-12, which contribute to decreasing oocyte quality [35]. Higher concentrations of inflammatory ILs observed in patients with endometriosis may contribute to the disruption of the oocyte spindle [32].

Stem cells have great potential as a therapy for various diseases. Stem cells have a broad regeneration capacity and the ability to produce daughter cells, which will undergo further differentiation. Bone marrow mesenchymal stem cells are adult stem cells derived from the bone marrow stroma. Mesenchymal stem cells can be used for cell therapy because they are easy to isolate, can be expanded widely without losing their differentiation ability, have low immunity, and are multipotent. The main role of mature stem cells is maintaining and repairing the tissue in which they are located [36]. Mesenchymal stem cells provide benefits through two mechanisms: (1) Mesenchymal stem cells differentiate to replace damaged cells to form new cytoarchitecture and (2) mesenchymal stem cells produce paracrine effects by releasing immunoregulators that support regeneration (tropic activity). One of the tropic activities of stem cells is inhibiting apoptosis.

The term “homing” can be defined as a process by which stem cells migrate and then stick firmly to the tissue. The distribution of stem cells to the tissues/organs is the first step that must be achieved so that stem cells can repair damaged tissues/organs. For that, we need a way so that the transplanted stem cells can reach the target tissue/organ [37].

In this study, to ensure that bone marrow mesenchymal stem cells reach the ovary as the target organ, they were stained and transplanted in all endometriosis mouse models. Two weeks after stem cell transplantation, the mice were euthanized, and their ovaries were removed. Mesenchymal stem cell colonization was analyzed using a fluorescence microscope [38].

TNF-α is an important cytokine regulator, which not only regulates the immune response but also influences cell differentiation, cell survival, and cell apoptosis. The effects caused by TNF-α depend on its binding to the receptor. TNF-α binding with death receptors (TNFR1) on the cell membrane will induce an apoptotic pathway. The ovaries of mice express both TNF-α receptors. In endometriosis, both types of receptors are also found in high concentrations.

Studies have shown that transplanting human amnion epithelial cells (hAECs) can reduce the production of TNF-α, IL-1, and IL-6. hAEC can reduce TNF-α, which is a mediator of apoptosis, thus, hAEC can inhibit apoptosis. The results of this study showed a decrease in the expression of TNFR1 (Table-6). This decrease causes the bond between TNFR1 and its ligands to weaken so that the apoptotic pathway does not work, which is prominent with a significant decrease in the number of apoptotic granulosa cells [39].

**Table-6:** The expression of TNF-α R1 in the control group, the endometriosis model group, and the endometriosis model group of mice given with mesenchymal stem cells.

Group	n	Mean±Standard deviation	p-value
T0 group	14	2.16±0.874^a^	0.004
T1 group	14	4.13±2.568^b^	
T2 group	14	1.73±1.481^a^	

*Different superscripts show significant differences. T0 group: Negative control group; T1 group: The positive control group; and T2 group: The treatment group

The decrease in the expression of TNFR1 in this study was only 19.5% against the incidence of apoptosis. This could be because mesenchymal stem cells also reduce the expression of their ligands (TNF-α) so that ligand and receptor binding does not occur, and then, the apoptotic pathway does not occur. This will interfere with folliculogenesis in terms of decreasing the number of pre-ovulatory follicles, the size of the dominant follicles, and the levels of estradiol. This is due to the increased apoptosis of granulosa cells in endometriosis [10].

Granulosa cells are responsible for steroidogenesis and oocyte maturation. Folliculogenesis is a process of follicular development in the ovaries, which involves several processes: Recruitment, selection, growth, maturation, and ovulation. The stages of folliculogenesis are divided into two. The first phase is the pre-antral phase or gonadotropin-independent phase, characterized by the growth and differentiation of the oocytes. The second phase is the antral phase or gonadotropin-dependent phase, characterized by an increase in the size of the follicle itself. The pre-antral phase is controlled primarily by locally produced growth factors through the autocrine/paracrine mechanism. Meanwhile, the antral phase is regulated by follicle-stimulating hormone (FSH) and luteinizing hormone (LH).

The dominant follicle is characterized by continuous granulosa cell mitosis. The selection of the dominant follicle begins at the end of the luteal phase of the previous cycle. FSH levels begin to rise near the end of the luteal phase, along with decreasing levels of progesterone and estrogen. *In vivo* and *in vitro* studies have shown that FSH directly stimulates human granulosa cell mitosis. Granulosa cells are the only cells that can express FSH receptors. The binding of FSH with its receptors in the transmembrane will cause the expression of the P450 aromatase gene and 17α-hydroxysteroid dehydrogenase, which contribute to the production of estradiol. P450 aromatase contributes to the conversion of androgen to estrogen. At the end of the follicular phase, the intrafollicular concentration of estradiol is directly related to follicle size and reaches a concentration of +1 μg/mL when the circulating estradiol level reaches its peak. Estradiol levels that reach their peak in the circulation will cause a surge in LH that triggers ovulation. FSH receptor signaling increases the expression of the LH receptor gene in granulosa cells. LH receptors cause granulosa cells in the dominant follicle to respond to an increase in LH mid-cycle and cause ovulation [40].

In bone marrow mesenchymal stem cell transplantation in endometriosis mouse models, the number of primary and secondary follicles was higher in the group that received stem cells, although not statistically different, whereas the number of tertiary follicles did not differ between groups with and without stem cells. Transplanting stem cells significantly increase the number of graafian follicles; therefore, stem cell transplantation can improve folliculogenesis by reducing the apoptosis of granulosa cells so that the steroidogenesis and oogenesis functions are back to normal and increase the ovulation rate.

## Conclusion

The transplantation of bone marrow mesenchymal stem cells in endometriosis mouse models can reduce the expression of TNFR1 and apoptosis of granulosa cells and improve folliculogenesis.

## Authors’ Contributions

SRD, FAD, and WW: Research concept and design. SRD and SD: Collection and assembly of data, SRD, HH: Data analysis and interpretation. SRD and WW: Writing the manuscript. EGD, SS and IWAW: Critical revision of the manuscript. All authors examined samples in the research laboratory. All authors compiled, read, revised, and approved the final manuscript.

## References

[1] Barnhart K, Dunsmoor-Su R, Coutifaris C (2002). Effect of endometriosis on *in vitro* fertilization. Fertil. Steril.

[2] Omland A.K, Abyholm T, Fedorcsák P, Ertzeid G, Oldereid N.B, Bjercke S, Tanbo T (2005). Pregnancy outcome after IVF and ICSI in unexplained, endometriosis-associated and tubal factor infertility. Hum. Reprod.

[3] Gupta S, Goldberg J.M, Aziz N, Goldberg E, Krajcir N, Agarwal A (2008). Pathogenic mechanisms in endometriosis-associated infertility. Fertil. Steril.

[4] Speroff L, Fritz M (2011). Endometriosis. Clinical Gynecologic Endocrinology and Infertility.

[5] Harada T, Enatsu A, Mitsunari M (1999). Role cytokine in progression of endometriosis. Gynecol. Obstet. Invest.

[6] Iwabe T, Harada T, Tsudo T, Tanikawa M (1998). Pathogenetic significance of increased levels of interleukin-8 in peritoneal fluid of patients with endometriosis. Fertil. Steril.

[7] Mahnke M.D, Jennifer L, Dawood Y (2000). Vascula endothelial growth factor and interleukin-6 in peritoneal fluid of women with endometriosis. Fertil. Steril.

[8] Craig A, Witz M.D (2000). Interleukin-6:Another piece of the endometriosis cytokine puzzle. Fertil. Steril.

[9] Bedaiwy M.A, Falcone T (2003). Peritoneal fluid environment in endometriosis, clinicopathological implication. Minerva Ginecol.

[10] Sanchez A.M, Somigliana E, Vercelini P, Pagliardhini L, Candiani M, Vigano P (2016). Endometriosis as a detrimental condition for granulosa cell steroidogenesis and development:From molecular alterations to clinical impact. J. Steroid Biochem. Mol. Biol.

[11] Taniguchi F, Adonakis G, Kaponis A, Harada T (2007). Apoptosis and endometriosis. Bioscience.

[12] Chen X, Wang Q, Li X, Wang Q, Xie J, Xiafei Fu X (2018). Heat shock pretreatment of mesenchymal stem cells for inhibiting the apoptosis of ovarian granulosa cells enhanced the repair effect on chemotherapy-induced premature ovarian failure. Stem Cell Res Ther.

[13] Na J, Gi Jin Kim G. J (2020). Recent trends in stem cell therapy for premature ovarian insufficiency and its therapeutic potential:a review. J Ovarian Res.

[14] Chi D.S, Harris N.S (1977). A simple method for the isolation of murine peripheral blood lymphocytes. J. Immunol. Methods.

[15] Ariffin S.H.Z, Kermani S, Megat A.W.R, Senafi S, Zainal A.Z, Abdul R.M (2012). *In vitro* chondrogenesis transformation study of mouse dental pulp stem cells. Sci. World J.

[16] Dickinson H, Milton P, Jenkin G (2012). The isolation and characterization of putative mesenchymal stem cells from the spiny mouse. Cytotechnology.

[17] Cheng C.C, Lian W.S, Hsiao F.S.H, Liu I.H, Lin S.P (2012). Isolation and characterization of novel murine epiphysis derived mesenchymal stem cells. PLoS One.

[18] Ghaneialvar H, Soltani L, Rahmani H.R, Lotfi A.S, Soleimani M (2018). Characterization and classification of mesenchymal stem cells in several species using surface markers for cell therapy purposes. Indian J. Clin. Biochem.

[19] Zhang T, Lee Y.W, Rui Y.F, Cheng T.Y, Jiang X.H, Li G (2013). Bone marrow-derived mesenchymal stem cells promote growth and angiogenesis of breast and prostate tumors. Stem Cell Res. Ther.

[20] Zuo W, Xie B, Li C, Yan Y, Zhang Y, Liu W, Huang J, Chen D (2018). The clinical applications of endometrial mesenchymal stem cells. Biopreserv. Biobank.

[21] Von Wolff M.I, Classen-Linke D.H, Krusche C.A, Beier-Hellwig K, Karl C, Beier H.M (1999). Tumour necrosis factor-a (TNF-α) in human endometrium and uterine secretion:An evaluation by immunohistochemistry, ELISA and semi-quantitative RT–PCR. Mol. Hum. Reprod.

[22] Siemieniuch M.J, Szóstek A.Z, Gajos K, Kozdrowski R, Nowak M (2016). Type of inflammation differentially affects expression of interleukin 1b and 6, tumor necrosis factor-a and toll-like receptors in subclinical endometritis in mares. PLoS One.

[23] Ueda M, Yamashita Y, Takehara M, Terai Y, Kumagai K, Ueki K, Kanda K, Yamaguchi H, Akise D, Hung Y.C, Ueki M (2002). Survivin gene expression in endometriosis. J. Clin. Endocrinol. Metab.

[24] Ilad R.S, Fleming S.D, Bebington C.R, Murphy C.R (2004). Ubiquitin is associated with the survival of ectopic stromal cells in endometriosis. Reprod. Biol. Endocrinol.

[25] Parasti R.V, Widjiati W, Dwiningsih S.R (2019). Effect of bone marrow mesenchymal stem cells transplantation on BMP-15 expression and graafian follicle count in mice model of endometriosis. Maj. Obs. Ginekol.

[26] Herlambang H, Rahman A.O, Kusdiyah E (2020). The effect of unripe dates (*Phoenix dactylifera*) on rat ovarian follicle maturation and ovulation. J. Kedokteran Brawijaya.

[27] Novak M, Madej J.A, Dziegeil P (2007). Intensity of Cox2 expression in cell of soft tissue fibrosarcomas in dogs as related to grade of tumour malignancy. Bull. Vet. Inst. Pulawy.

[28] Schmelzer E, McKeel D.T, Gerlach J.C (2019). Characterization of human mesenchymal stem cells from different tissues and their membrane encasement for prospective transplantation therapies. Biomed Res. Int.

[29] Karaoz E, Aksoy A, Ayhan S, Sarıboyacı A.E, Kaymaz F, Kasap M (2009). Characterization of mesenchymal stem cells from rat bone marrow:Ultrastructural properties, differentiation potential and immunophenotypic markers. Histochem. Cell Biol.

[30] Dinaryanti A, Karsari D, Ertanti N, Ihsan I, Ariyanti A, Rantam F.A, Aulanni'am, Purwati (2019). Isolation and characterization of skin derived mesenchymal stem cell (Smscs) from New Zealand rabbit, *Oryctolagus cuniculus*:A *in vitro* study. Biochem. Cell. Arch.

[31] Sanchez A.M, Vanni V.S, Bartiromo L, Papaleo E, Zilberberg E, Candiani M, Orvieto R, Viganò P (2017). Is the oocyte quality affected by endometriosis?A review of the literature. J. Ovarian Res.

[32] Sanchez A.M, Somigliana E, Vercellini P, Pagliardini L, Candiani M, Vigano P (2016). Endometriosis as a detrimental condition for granulosa cell steroidogenesis and development:From molecular alterations to clinical impact. J. Steroid Biochem. Mol. Biol.

[33] Giacomini E, Sanchez A.M, Sarais V, Beitawi S.A, Candiani M, Viganò P (2017). Characteristics of follicular fluid in ovaries with endometriomas. Eur. J. Obstet. Gynecol. Reprod. Biol.

[34] Da Broi M.G, de Albuquerque F.O, de Andrade A.Z, Cardoso R.L, Jordão A.A. Jr, Navarro P.A (2016). Increased concentration of 8-hydroxy-2'-deoxyguanosine in follicular fluid of infertile women with endometriosis. Cell Tissue Res.

[35] Wu G, Bersinger A.N, Mueller M.D, von Wolff M (2017). Intrafollicular inflammatory cytokines but not steroid hormone concentrations are increased in naturally matured follicles of women with proven endometriosis. J. Assist. Reprod. Genet.

[36] Bongso A, Lee E.H, Bongso A, Lee E.H (2005). Stem Cells:From Bench to Bed Side.

[37] Hendarto H, Komarhadi M, Darmawanti E, Wijiati W, Suhatno S (2013). The effect of bone marrow transplantation on oocyte-granulosa cell interaction and follicular development of cisplatin-induced ovarian failure in rat. J. Stem Cell Res. Ther.

[38] Barminco J, Gray A, Maguire T, Schloss R, Yarmush M.L, Chase L.G, Vemuri M.C (2013). Mesenchymal stromal cell mechanisms of immunomodulation and homing. Mesenchymal Stem Cell Therapy, Stem Cell Biology and Regenerative Medicine.

[39] Zhang Q, Xu M, Yao X, Li T, Wang Q, Lai D (2015). Human amniotic epithelial cells inhibit granulosa cell apoptosis induced by chemotherapy and restore the fertility. Stem Cell Res. Ther.

[40] Conti M, Chang J.R (2016). Folliculogenesis, Ovulation and Luteogenesis. Endocrinology:Adult and Pediatric.

